# Fungal literature records database of the Northern West Siberia (Russia)

**DOI:** 10.3897/BDJ.8.e52963

**Published:** 2020-07-09

**Authors:** Nina Filippova, Stanislav Arefyev, Elena Zvyagina, Vladimir Kapitonov, Tatiana Makarova, Victor Mukhin, Nellya Sedelnikova, Iraida Stavishenko, Anton Shiryaev, Tatiana Tolpysheva, Natalia Ryabitseva, Alexander Paukov

**Affiliations:** 1 Yugra State University, Khanty-Mansiysk, Russia Yugra State University Khanty-Mansiysk Russia; 2 Institute of the Problems of Northern Development of Tyumen Scientific Centre SB RAS, Tyumen, Russia Institute of the Problems of Northern Development of Tyumen Scientific Centre SB RAS Tyumen Russia; 3 Federal Nature Reserve "Yuganskiy", Ugut, Russia Federal Nature Reserve "Yuganskiy" Ugut Russia; 4 Surgut State University, Surgut, Russia Surgut State University Surgut Russia; 5 Tobolsk Complex Scientific Station of the UrB RAS, Tobolsk, Russia Tobolsk Complex Scientific Station of the UrB RAS Tobolsk Russia; 6 Ural Federal University, Ekaterinburg, Russia Ural Federal University Ekaterinburg Russia; 7 Central Siberian Botanical Garden, Novosibirsk, Russia Central Siberian Botanical Garden Novosibirsk Russia; 8 Institute of Plant and Animal Ecology of Ural branch of RAS, Ekaterinburg, Russia Institute of Plant and Animal Ecology of Ural branch of RAS Ekaterinburg Russia; 9 Moscow State University, Moscow, Russia Moscow State University Moscow Russia

**Keywords:** occurrence, specimen, funga, Mycobiota, digitisation, data mobilisation

## Abstract

**Background:**

Mycological research in the Northern part of West Siberia has now become sufficient for review and digitisation as over 460 scientific works have been completed mainly since the beginning of the 20th century. The history of research in the region started from isolated studies at the beginning of the 20th century, but regular and systematic research started from the 1970s. Over the following decades, several dozens of researchers have worked in the area, but the reported occurrences were scattered amongst a broad variety of publications, mainly hardly available. The great need in digitisation and accumulation of fungal records reported in published literature in a standardised regional database has now become evident. The «Fungal records database of the Northern West Siberia» (FuNWS) was initiated in 2016 according to contemporary biodiversity data standards (Darwin Core), to be compatible and accessible by the broad research community. The database has been supplemented ever since by the collective effort of specialists working in the area. According to the database summary report, there are 3358 fungal and fungus-like species revealed in the Northern West Siberia at present. The richest in species number classes are Agaricomycetes (60%) and Lecanoromycetes (33%) with a total of 25 classes represented. The FuNWS database was uploaded to Global Biodiversity Information Facility (GBIF) (Ygra State University Biological Collection publisher) on 11 November 2017 (earlier titled «Fungal Records Database of Yugra, FReDY») to provide open access to the data and its reusability (Filippova et al. 2020).

**New information:**

This publication summarises the results of the digitisation of literature-based occurrence records of fungi and fungus-like organisms initiated in the Northern part of West Siberia for the first time in the history of mycological research. The bibliography of regional mycological publications was created to include about 460 published works (Suppl. material 2). In total, about 140 literature sources were digitised and about 22000 occurrence records were integrated into the FuNWS database (Filippova et al. 2020).

## Introduction

The mycological research in the Northern part of West Siberia stems from isolated studies in the beginning of the 20th century, yet regular and systematic research only began in the second half of the century. Over the following decades, several dozen researchers worked in the area and a total of over 460 scientific works were published. The history of mycological research in the southern half of this area was described in two publications (Filippova et al. 2017b, Filippova et al. 2017a). The history of research of particular groups of fungi was reviewed in corresponding monographs and regional checklists (Magomedova and Ektova 2006, Magomedova et al. 2006, Mukhin 1993, Karatygin et al. 1999). The common checklist of fungi for the total area of the Northern West Siberia does not yet exist and the species occurrences were scattered amongst a broad variety of publications, mainly hardly available. The biodiversity data digitisation and mobilisation programme started in the region recently, bringing the standard approach to biodiversity data storage and their integration into common portals. In line with this programme, we carried out digitisation of literature-based occurrences of fungi reported in the region. A database of occurrence records was created to accumulate those extracted from literature records, which could be considered as a substitute for printed checklists or funga of older times.

The database was initiated in 2016 using Google Sheets (a web-based service, https://www.google.com/sheets/about/) as a table formatted in accordance with the Darwin Core standards (Filippova and Bolshakov 2017). The species occurrence records were filled in by the collective effort of specialists working in the area. The first published version of the database was dedicated solely to the Khanty-Mansi Autonomous Okrug – Yugra in its administrative borders (Filippova and Bulyonkova 2018). Additional literature was added later to cover the whole Northern West Siberia (including two administrative regions: Yugra and Yamalo-Nenets Autonomous Okrug) and the database was re-named accordingly (Filippova et al. 2020).

According to the database summary report, there are about 3358 species identified in the region to date. Amongst 25 classes represented in the data, the richest are Agaricomycetes (60%) and Lecanoromycetes (30%).

Below we describe the history of mycological research in the Northern part of West Siberia in each administrative region by traditionally-studied morphological or ecological groups.

### Overview of the mycological research reflected in the database

**Yamalo-Nenets Autonomous Okrug**

About 25 researchers participated in the inventory of **lichens** in the region. The most complete species lists were published in a series of works (Andreev 1982, Andreev 1984, Ahmet'ev et al. 1993, Magomedova and Ektova 2006, Magomedova et al. 2006, Pristyazhnyuk 1994, Pristyazhnyuk 1996, Pristyazhnyuk 1998, Pristyazhnyuk 2001, Ryabkova 1998, Sedelnikova 2017, Zhurbenko 1999). The history of the inventory of lichens in the region was described in detail for the Urals in Ryabkova (1965), for the Yamal Peninsula in Magomedova and Ektova (2006) and for the Polar Urals in Magomedova et al. (2006). In the first half of the 20th century, studies of reindeer husbandry and productivity of lichens were initiated by K. N. Igoshina in a series of works Igoshina (1933), Igoshina (1935), Igoshina (1937), Igoshina (1939), Igoshina and Frolovskaya (1939). The assessment of natural factors, as well as grazing and pyrogenic factors on lichens productivity, was continued later in the Polar Urals in a series of publications (Abdulmanova and Ektova 2015b, Abdulmanova and Ektova 2015a, Abdulmanova and Ektova 2013, Ektova and Morozova 2015).

**Agaricoid basidiomycetes** is a less-studied group in the Yamalo-Nenets Autonomous Okrug compared to the bordering southern region. Sporadic studies were conducted in the Polar Urals by Kazantseva (1966), Kazantseva (1968b), Kazantseva (1970), Knudsen and Mukhin (1998), in the Southern Yamal by Tarchevskaya (1985a), Tarchevskaya (1985b), Tarchevskaya (1986), Tarchevskaya (1990) and in the Tazovskiy peninsula by Kapitonov (2015). Regular inventories and herbarium collections were conducted at several field stations of the Komarov Botanical Institute of the Russian Academy of Sciences working in the region in the second half of the 20th century (Karatygin et al. 1999). The collections made during this period are stored in the LE herbarium (Saint-Petersburg) and later processed in a series of publications (Kovalenko 1999, Malysheva 2018, Nezdoiminogo 1996, Nezdojminogo 2001).

**Clavarioid basidiomycetes** are a well-studied group mainly by a single researcher working in different regions: the Polar Urals (Shiryaev 2006), Novaya Zemlya, Yamal, Beliy island and Gydana (Shiryaev 2011) and in the Middle Urals (Shiryaev 2004). The geographical distribution of the clavarioid fungi was analysed in a number of works (Shiryaev 2013, Shiryaev et al. 2016, Shiryaev 2017, Shiryaev 2018). The impact of climate change on the clavarioid fungi is hypothesised in several papers (Shiryaev 2009, Shiryaev et al. 2019).

**Lignicolous basidiomycetes** are a well-studied ecological group in the North of West Siberia. N. T. Stepanova-Kartavenko initiated the inventory of the middle Urals (Stepanova-Kartavenko 1967) and made some works in the Polar Urals (Stepanova and Sirko 1970). L. K. Kazantseva dedicated the study of wood-decay mycobiota to the northern regions of the Polar Urals and Yamal (Kazantseva 1971a, Kazantseva 1971b, Kazantseva 1972). V. A. Mukhin analysed the biogeography and ecology of lignicolous basidiomycetes in West Siberia, from the forest-steppe zone in the South to the tundra-steppe in the North (Mukhin 1984, Mukhin 1987a). The same author examined the local mycobiotas in several publications (Mukhin and Stepanova 1982, Mukhin and Stepanova 1983, Mukhin 1983, Mukhin 1987b, Mukhin 1991, Mukhin and Olshvang 1983). S. P. Arefyev studied lignicolous communities on imported wood in the Yamal Peninsula (Arefyev 2002) and made a revision of the lignicolous community in the Verzhne-Tazovskiy Nature Reserve (Arefyev 2004). He also initiated important research of lignicolous communities and their transformation in the urban centres of the North (Arefyev 1996, Arefyev 1998).

A number of works was performed to study **fungal pathogens** of plants in the region, by Demidova (1962), Demidova (1970), Kazantseva (1968a) and Stepanova (1970). Some records of pathogens of cereals are reported in the monograph by Lavrov (1951) on the mycoflora of cereals of Siberia. A series of inventories performed at the former field stations of the Komarov Botanical Institute (collections stored in LE) were summarised in Karatygin et al. (1999).

**Myxomycetes** of the Urals, including its northern territories, are described in the PhD thesis by Fefelov (2006) and collections stored in LE are summarised in Karatygin et al. (1999).

**Soil microfungi** were studied in a few works (Kulay 1968, Kulay and Ischenko 1974), as well as in the PhD thesis by Ischenko (1981).

The occurrence records of **discomycetes and other ascomycetes** appeared in the papers by A. V. Raitvir with co-authors (Raytviyr and Sirko 1968, Sirko 1970a, Sirko 1970b, Sirko 1971, Kazantseva and Sirko 1974), with collections stored in LE being summarised in Karatygin et al. (1999).

Additionally, B. V. Krasutsky was deeply engaged in the ecological study of **fungivorous Coleoptera** communities (Krasutskiy 2007), inventorying several localities in the region.

**Khanty-Mansi Autonomous Okrug**

**Lignicolous basidiomycetes** have been studied quite extensively by a number of researchers. S. P. Arefyev initiated regional studies on wood-pathogens (Arefyev 1990, Arefyev 1991) and applied ecological modelling of lignicolous communities (Arefyev 2003, Arefyev 2008b, Arefyev 2010). Along with these approaches, the same author inventoried several regions and protected areas (Arefyev 2003, Arefyev 2008a, Arefyev 2011, Arefyev 2013). V. A. Mukhin (1993) analysed the lignicolous communities along a latitudinal gradient in Western Siberia in a comprehensive monograph. I. V. Stavishenko contributed greatly to the knowledge of species diversity in the regional conservation areas (Stavishenko 2000, Stavishenko 2003, Stavishenko 2007a, Stavishenko 2007b, Stavishenko 2011, Stavishenko and Mukhin 2002, Stavishenko and Zalesov 2008). Some recommendations on monitoring of lignicolous fungi in protected areas (Stavishenko 1996, Stavishenko 1997, Stavishenko 2008) and in oil and gas production areas (Stavishenko and Zalesov 2008) were developed.

The inventories of **lichens** were performed in a number of protected areas in the region, with the highest number of species revealed in the Polar Urals and adjacent areas (Paukov and Mikhaylova 2011, Ryabkova et al. 1996, Sedelnikova and Taran 2000, Sedelnikova 2010, Tolpysheva and Shishkonakova 2019, Trapeznikova 2003, Shalatonov 2010, Chabanenko and Taran 2004). Attention was paid to the restoration processes of lichen cover in disturbed areas (Shishkonakova and Tolpysheva 2018, Tolpysheva and Shishkonakova 2020, Shishkonakova et al. 2013) and under natural regression of peatlands (Shishkonakova et al. 2016). Several papers were devoted to the lichens of raised bogs, covering large areas in the region (Lapshina and Koneva 2010, Tolpysheva 2004).

**Marcofungi** were studied in a number of areas, but the most thoroughly studied area were centred around Khanty-Mansiysk and, in the south-east part, in and near the Yuganskiy Nature Reserve. The Nature Reserve has been inventoried since 2007 in a number of studies (Zvyagina et al. 2009, Zvyagina, E.A. and Baykalova, A.S. 2017, Zvyagina et al. 2007, Zvyagina 2012, Zvyagina 2015). In the Khanty-Mansiysk vicinity, the inventory was targeting particular communities of peatlands and forests (Filippova and Thormann 2014, Filippova and Bulyonkova 2017, Filippova et al. 2015). The permanent plot-based monitoring of macromycetes fruiting dynamics has been initiated since 2014 in different vegetation types (Filippova et al. 2014, Filippova and Bulyonkova 2017). Some other protected areas of Yugra were visited by other researchers and the checklists were published (Zvyagina and Vasina 2015, Kapitonov 2012, Makarova et al. 2015, Shiryaev 2002).

The study of the diversity of **myxomycetes** was carried out in two protected areas (Fefelov 2002, Fefelov 2007). The community of corticolous myxomycetes was sampled nearby Khanty-Mansiysk with the description of two new species (Vlasenko et al. 2019, Vlasenko et al. 2018).

Phytopathological studies are developing in the city of Surgut. The flora of **fungal pathogens** of the city parks of Surgut was studied for many years by T. A. Marakova and colleagues (Makarova et al. 2011, Makarova and Makarov 2016).

The communities of **microfungi** and yeasts were sampled in a study of mycobiota of raised bogs (Filippova 2012, Filippova 2015, Filippova and Thormann 2015, Kachalkin 2010). Some works were devoted to the study of the influence of lichens on soil micromycetes (Tolpysheva 2006).

## General description

### Purpose

This is the first example of digitisation of species occurrence data published in literature in the Northern part of West Siberia and its publication as a GBIF dataset. The paper also provides the contemporary analysis of the research state of the funga in the region. The aim of the data paper was to provide the description and the link to the published dataset in the format of a peer-reviewed journal paper and to provide recognition for the effort by means of a scholarly article (based on Data paper definition published at https://www.gbif.org/en/data-papers).

## Project description

### Title

Biodiversity data digitisation and mobilisation in Northern West Siberia (https://nwsbios.org)

### Personnel

Nina Filippova

## Sampling methods

### Study extent

The digitisation was aimed at summarising the species occurrences of fungi and fungi-related organisms accumulated in the course of previous mycological studies and published in peer-reviewed scientific literature. The geography extended to the Northern part of West Siberia, in the administrative borders of two regions (Yamalo-Nenets Autonomous Okrug and Khanty-Masi Autonomous Okrug-Yugra). Over 460 publications were reviewed and the species occurrence records were extracted from about 140 selected works. About 80% of species occurrences accumulated in the database were relatively recent, i.e. published in literature since the beginning of 21st century.

### Sampling description

Methods of sampling vary in different reviewed publications, but generally follow the protocols of Mueller et al. (2004) for different taxonomical and ecological groups. The majority of the records were made using direct observation of fruiting structures. The exception are a few studies of micromycetes and yeasts where cultivation techniques were applied. No molecular (environmental sampling) methods were used until the present to reveal molecular diversity of fungi in the region. Plot-based monitoring of terrestrial and lignicolous macrofungi was organised by some researchers, providing estimates of quantitative parameters and temporal dynamics of fungal communities. The majority of fungal occurrence records were accompanied by accessioning of specimens in fungaria, although the specimen numbers are rarely reported in publications. The specimens are stored in different collections within and outside the region where the researchers were working (i.e. the main collections of LE - the Komarov Botanical Institute, Saint-Petersburg; SVER - Institute of Ecology of Animals and Plants, Ekaterinburg; NSK - Central Siberian Botanical Garden, Novosibirsk and others).

### Quality control

The original species identifications from the published works were recorded in the database, although no attempt was made for the revision of the species identification accuracy. A single author revised the species list and corrected wrong original identifications: the corrected names were added in identificationRemarks field, totalling 15 records in the database. The incorrect spelling of taxa was verified using the GBIF Species Matching tool at the later stage of the database compilation. Possible georeference errors at the scale of the region were corrected using QGIS software (https://qgis.org/en/site) by eliminating the outliers. Depending on the quality of georeferences provided in publications, the uncertainty was estimated as follows: 1) the coordinate of a fruiting structure or a plot provided in the publication gave the uncertainty about 3-10 m; 2) the coordinates of the fieldwork locality provided in publications gave the uncertainty to about 500 m – 5 km; 3) the report of the species presence in the district or the region gave the central coordinates of the area with the uncertainty radius to include its borders. The occurrences with large uncertainties were not eliminated, as they can still be important in the global context.

### Step description

1. The bibliography of related publications was compiled using Zotero bibliographic manager (https://www.zotero.org). Only published works (peer-reviewed papers, conference proceedings, PhD theses, monographs or book chapters) were selected.
2. The layout of the FuNWS database was made using Google Sheets software. Such database could be filled simultaneously by several specialists and a common data format will be provided (Filippova and Bolshakov 2017).
3. The Darwin Core standard was applied to the database structure to accommodate the relevant information extracted from the publications.
4. From the available bibliography of publications related to the region, only works with species occurrences were selected for the databasing purpose. We decided to include all different sorts of occurrence records, from a mere mention of the species within the administrative region, to the annotated species lists with exact locations of the records.
5. All occurrence records were georeferenced, either from the coordinates provided in the paper or from the verbal description of the fieldwork locality. The georeferencing of the verbal descriptions was made using Yandex (https://yandex.ru/maps) or Google (https://maps.google.ru/maps) maps services.
6. The coordinate uncertainty was estimated according to the algorithm described above (see Quality Control paragraph).
7. The locality names reported in Russian were translated into English and written in the «locality» field. Russian descriptions were reserved in the field «verbatimLocality» for accuracy.
8. When possible, the «eventDate» was extracted from the species records annotation data. Whenever this information was absent, the date of the publication was used instead.
9. The ecological features, habitat and substrates preferences were written in the «habitat» and reserved in Russian.
10. The original scientific names, reported in publications, were filled in the «originalNameUsage». Correction of the spelling errors was made using the GBIF Species Matching tool.
11. The GBIF Species matching tool was used to create the additional fields of taxonomic hierarchy from species to kingdom, to fill in the «taxonRank» field and to make synonymisation according to the GBIF backbone.
12. The taxonomic and spatial analyses of the final database were made using Microsoft Excel, QGIS and R software (https://www.r-project.org).
13. To track the digitisation process, a working database was created. Each bibliographic record has a series of fields to describe the digitisation process and its results: the total number of extracted occurrence records, general description of the occurrence quality, presence of observation date, presence of specimen number and details of georeferencing (Suppl. material 1).

## Geographic coverage

### Description

The dataset is limited by the administrative borders of two regions (Yamalo-Nenets Autonomous Okrug, Khanty-Masi Autonomous Okrug-Yugra). However, in cases where the digitised work contained records from other regions, they were also entered into the database (totalling about 1300 such records). The region occupies the central to Northern part of the West Siberian Plain. The area extends for about 1300 km from the West to the East, from the Eastern slopes of the Ural mountains to Yenisey river and from North to South – about 1600 km. The total area equals about 1,300,000 km^2^.

The relief of the region is mainly a plain, but the western part of the area is occupied by the Ural mountains with the highest points reaching up to 2000 m. The three vegetation zones (taiga, forest-tundra and tundra) and well-developed peatland cover represent the plain, while the mountain vegetation of the Urals changes from taiga to alpine zones.

In the southern half of the area (Yugra region), most administrative divisions were covered by mycological research, but the intensity of the research varied. A total of 80% of all records in the database have been made from four districts (Khanty-Mansiyskiy, Surgutskiy, Berezovskiy, Sovetskiy). In total, about 13000 records or 60% come presently from the Yugra region.

The northern part of the region was represented by less numbers of records in the database (about 6000 or 27%). The research is mainly concentrated in two districts (Priuralskiy - 66% of records and Yamalskiy - 22%).

**Figure 1.**
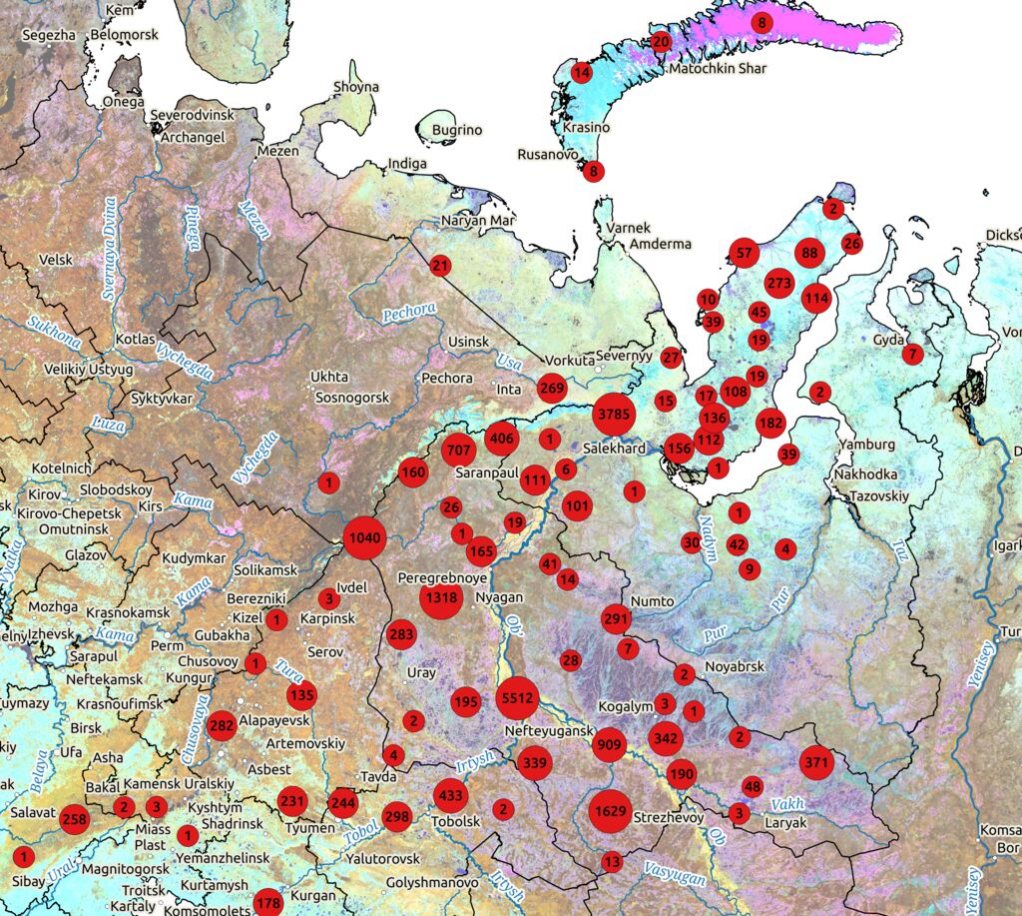
The distribution of the occurrence records from the FuNWS on Landsat satellite image of the area. The clustering of points was made within a radius of 50 km; the scale breaks were selected manually after plotting the frequency distribution histogram.

Generally, localities of the studies are situated randomly, with no attempts for regular studies using grid pattern having been made before. The areas under different kinds of nature protection are better studied compared to others: about half of all records in the database come from 13 protected areas (Fig. 1).

### Coordinates

58.309 and 73.749 Latitude; 58.887 and 86.353 Longitude.

## Taxonomic coverage

### Description

According to the database summary report, there are about 3358 species revealed in Northern West Siberia to date, representing 1020 genera, 293 families, 94 orders, 25 classes, six phyla and two kingdoms (Fungi, Protozoa). The richest studied classes by number of occurrences are Agaricomycetes (60%) and Lecanoromycetes (30%). The richest ten families by number of species are Parmeliaceae (144 species), Russulaceae (111), Physciaceae (99), Cortinariaceae (96), Tricholomataceae (93), Polyporaceae (84), Lecanoraceae (83), Cladoniaceae (81), Hymenogastraceae (79) and Ramalinaceae (67 species).

### Taxa included

**Table:** 

Rank	Scientific Name	
kingdom	Fungi	
kingdom	Protozoa	

## Temporal coverage

### Notes

1905-01-01 through 2020-01-01

## Usage rights

### Use license

Other

### IP rights notes

This work is licensed under a Creative Commons Attribution (CC-BY) 4.0 License.

## Data resources

### Data package title

Fungal literature records database of the Northern West Siberia (Russia)

### Resource link

https://www.gbif.org/dataset/29e78377-34c3-4c91-8062-550069a92b70

### Alternative identifiers

https://doi.org/10.15468/hfje3l; http://gbif.ru:8080/ipt/resource?r=fredy;

### Number of data sets

1

### Data set 1.

#### Data set name

Fungal literature records database of the Northern West Siberia (Russia)

#### Data format

Darwin Core

#### Number of columns

25

#### Description

The dataset includes a table in Darwin Core format with 28 fields and about 22000 records.

**Data set 1.** 

Column label	Column description
occurrenceID	https://dwc.tdwg.org/terms/#dwc:occurrenceID; an identifier of a particular occurrence, unique within this dataset. An abbreviation in the identifier' number (FReDY-xxxxxx) inherited from the previous name of the dataset (Fungal Records Database of Yugra).
bibliographicCitation	https://dwc.tdwg.org/terms/#dcterms:bibliographicCitation; the bibliographic citation of a publication from which the occurrence was extracted.
scientificName	https://dwc.tdwg.org/terms/#dwc:scientificName; the original names as provided in publication, but corrected for spelling mistakes using GBIF Species Matching tool.
verbatimLocality	https://dwc.tdwg.org/terms/#dwc:verbatimLocality; the original locality description of the collection place below county level, in Russian.
locality	https://dwc.tdwg.org/terms/#dwc:locality; the locality description translation in English.
habitat	https://dwc.tdwg.org/terms/#dwc:habitat; the description of habitat, including vegetation and substrate, in Russian or English.
fieldNumber	https://dwc.tdwg.org/terms/#dwc:fieldNumber; the herbarium or field specimen number, when reported in the source.
basisOfRecord	https://dwc.tdwg.org/terms/#dwc:basisOfRecord
year	https://dwc.tdwg.org/terms/#dwc:year; the year of observation/collection, if provided in publication. If no particular date were reported, the year of the publication itself was applied as the observation date.
month	https://dwc.tdwg.org/terms/#dwc:month; the month of observation/collection, if provided in publication.
day	https://dwc.tdwg.org/terms/#dwc:day; the day of observation/collection, if provided in publication.
countryCode	https://dwc.tdwg.org/terms/#dwc:countryCode
stateProvince	https://dwc.tdwg.org/terms/#dwc:stateProvince; the administrative unit below Country level (Okrug, Oblast, Respublica, Kray).
county	https://dwc.tdwg.org/terms/#dwc:county; the administrative unit below stateProvice level (Rayon).
decimalLatitude	https://dwc.tdwg.org/terms/#dwc:decimalLatitude
decimalLongitude	https://dwc.tdwg.org/terms/#dwc:decimalLongitude
coordinateUncertaintyInMeters	https://dwc.tdwg.org/terms/#dwc:coordinateUncertaintyInMeters; see "Quality control" chapter for the description of the uncertainty calculation algorithm.
geodeticDatum	https://dwc.tdwg.org/terms/#dwc:geodeticDatum
georeferenceSources	https://dwc.tdwg.org/terms/#dwc:georeferenceSources; the resource used to georeference the locality (Yandex maps, Google maps or georeferenced in publication).
taxonRank	https://dwc.tdwg.org/terms/#dwc:taxonRank; extracted from GBIF using Species Matching tool.
kingdom	https://dwc.tdwg.org/terms/#dwc:kingdom; extracted from GBIF using Species Matching tool.
eventDate	https://dwc.tdwg.org/terms/#dwc:eventDate; the full date of the observation event if provided in publication or the year of publication itself.
identificationRemarks	http://rs.tdwg.org/dwc/terms/identificationRemarks; comments or notes about the identification or missing taxa in GBIF backbone.
identificationQualifier	https://dwc.tdwg.org/terms/#dwc:identificationQualifier; a standard term ("cf.", "aff.") to express the determiner's doubts about the Identification.
language	https://dwc.tdwg.org/terms/#dcterms:language; languages used to describe the different fields of a record.

## Supplementary Material

8959A285-E8AC-5426-B5A6-A245109773BD 10.3897/BDJ.8.e52963.suppl1 Supplementary material 1 The digitization database Data type Bibliography Brief description To track the digitisation process, a working database was created. Each bibliographic record has a series of fields to describe the digitisation process and its results: the total number of extracted occurrence records, general description of the occurrence quality, presence of observation date, presence of specimen number and details of georeferencing. File: oo_423799.txt https://binary.pensoft.net/file/423799 Filippova, N.V.

44EC8312-D9B8-5B6D-80BB-E5E01AAC463E 10.3897/BDJ.8.e52963.suppl2 Supplementary material 2 The bibliography of mycological research in the Northern West Siberia Data type Bibliography Brief description The bibliography presents all scientific publications (journal papers, conference proceedings, PhD theseses, monographs and book chapters). The bibliography of publications was formatted according to the rules of Scopus: transliteration and translation of all Russian-language sources was made for the convenience of a foreign reader and standardisation of citations in English-language publications. File: oo_423800.txt https://binary.pensoft.net/file/423800 Filippova, N.V.
